# Supplementary material for: Heat stress causes economic and welfare disparities across agroecological zones in Burkina Faso
Source: Commun Earth Environ. 2025 Sep 9;6(1):744. doi: 10.1038/s43247-025-02650-1 (PMC12420375; doi:10.1038/s43247-025-02650-1)
Supplement: Supplementary file 2 — Supplementary Information [file 43247_2025_2650_MOESM2_ESM.pdf]

Supplementary information

Supplementary Figure 1. Heat stress-induced labour productivity changes under 1.5°C and 3.5°C warming (in %)

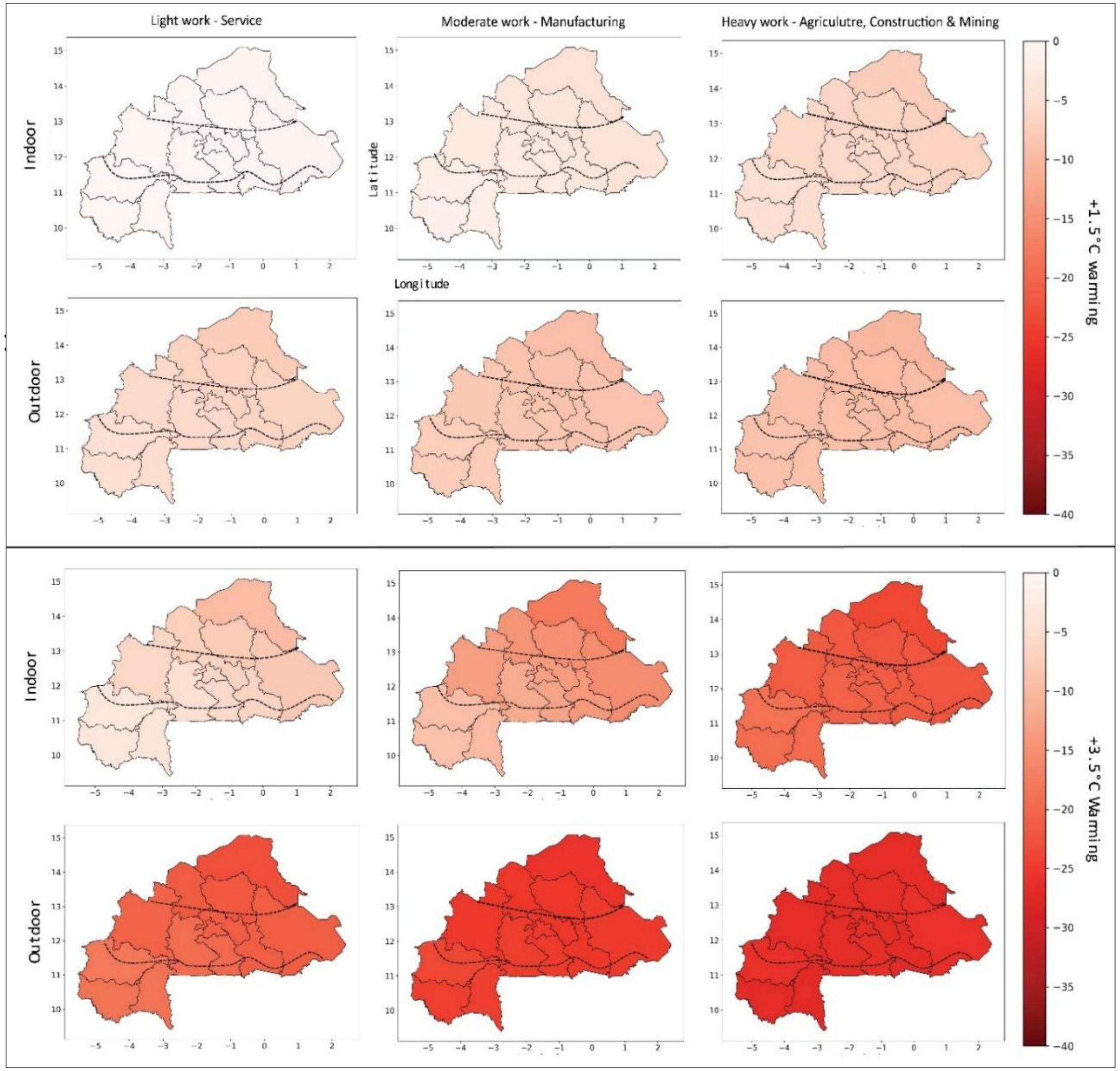

Supplementary Figure 2. The effects of heat stress-induced labour productivity loss on the use of intermediate inputs

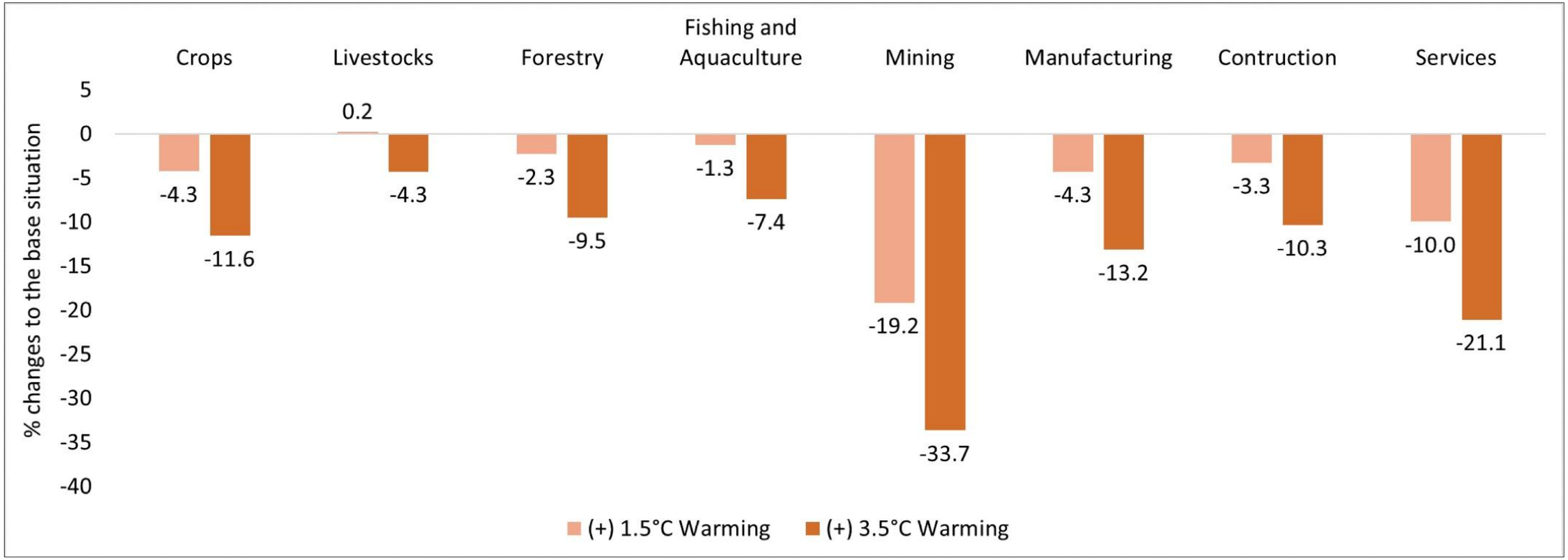

Supplementary Figure 3. The effects of heat stress-induced labour productivity loss on livestock across AEZs

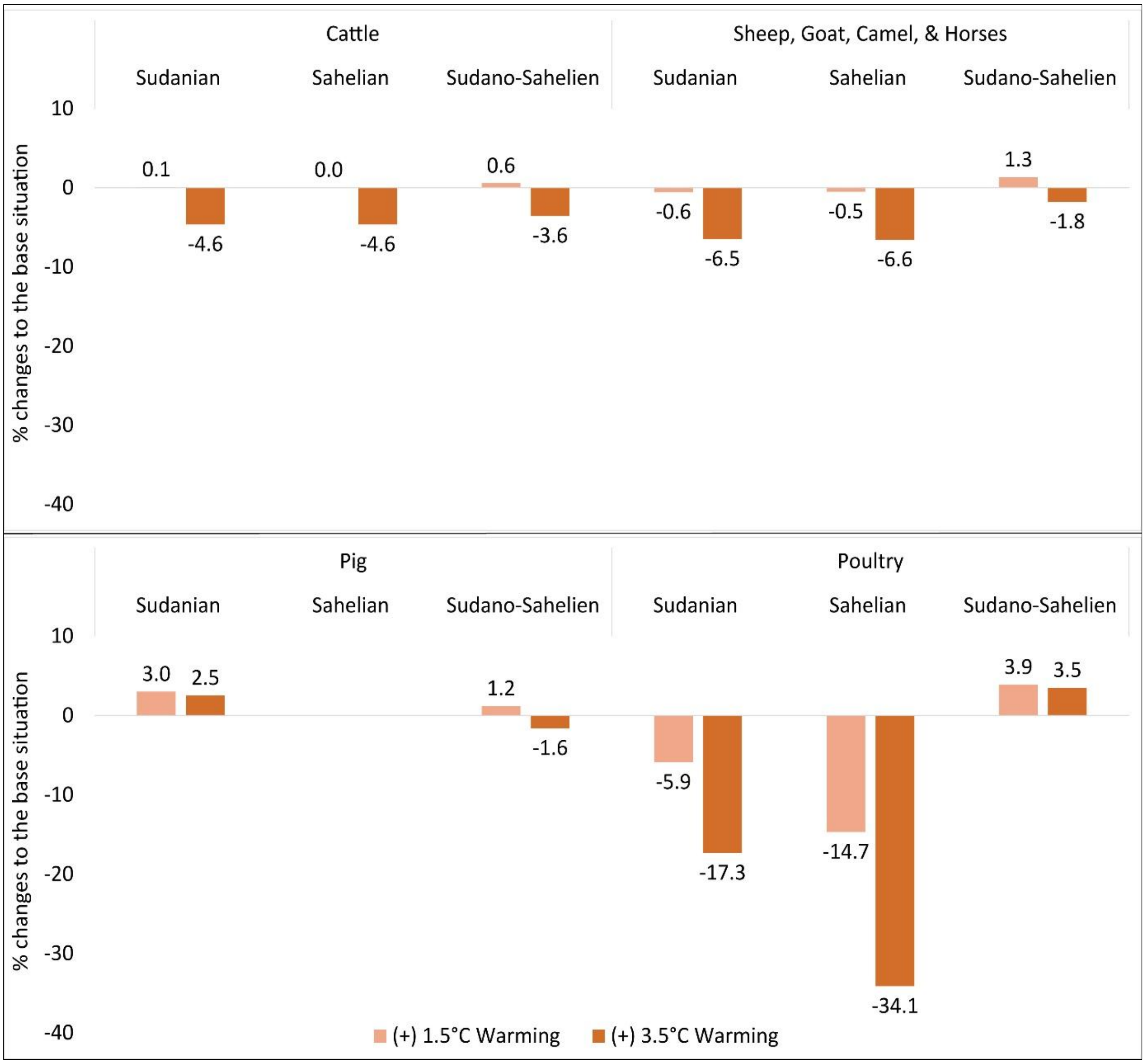

Supplementary Figure 4. The effects of heat stress-induced labour productivity loss on household income

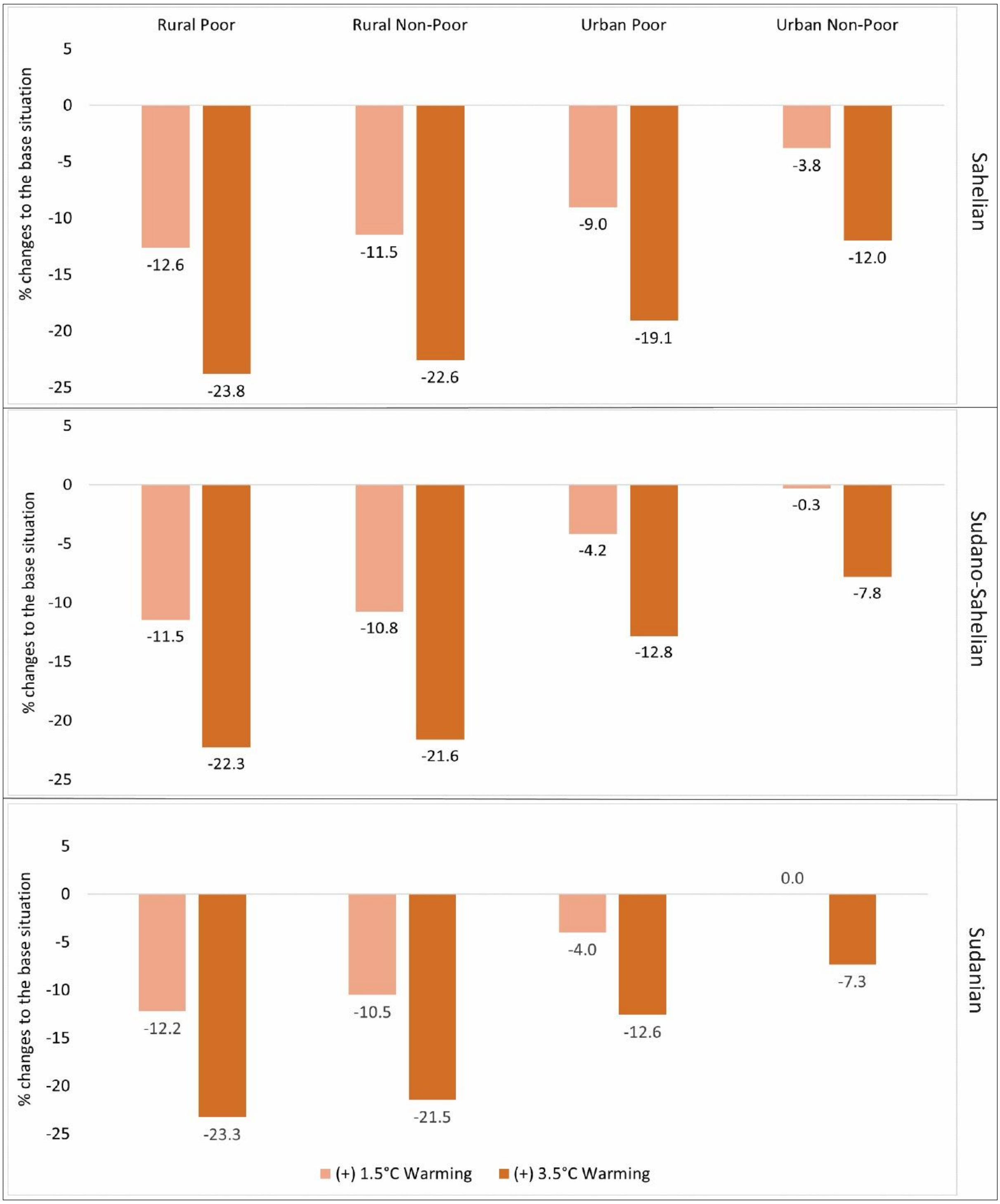

Supplementary Figure 5. The effects of heat stress-induced labour productivity loss on consumption prices

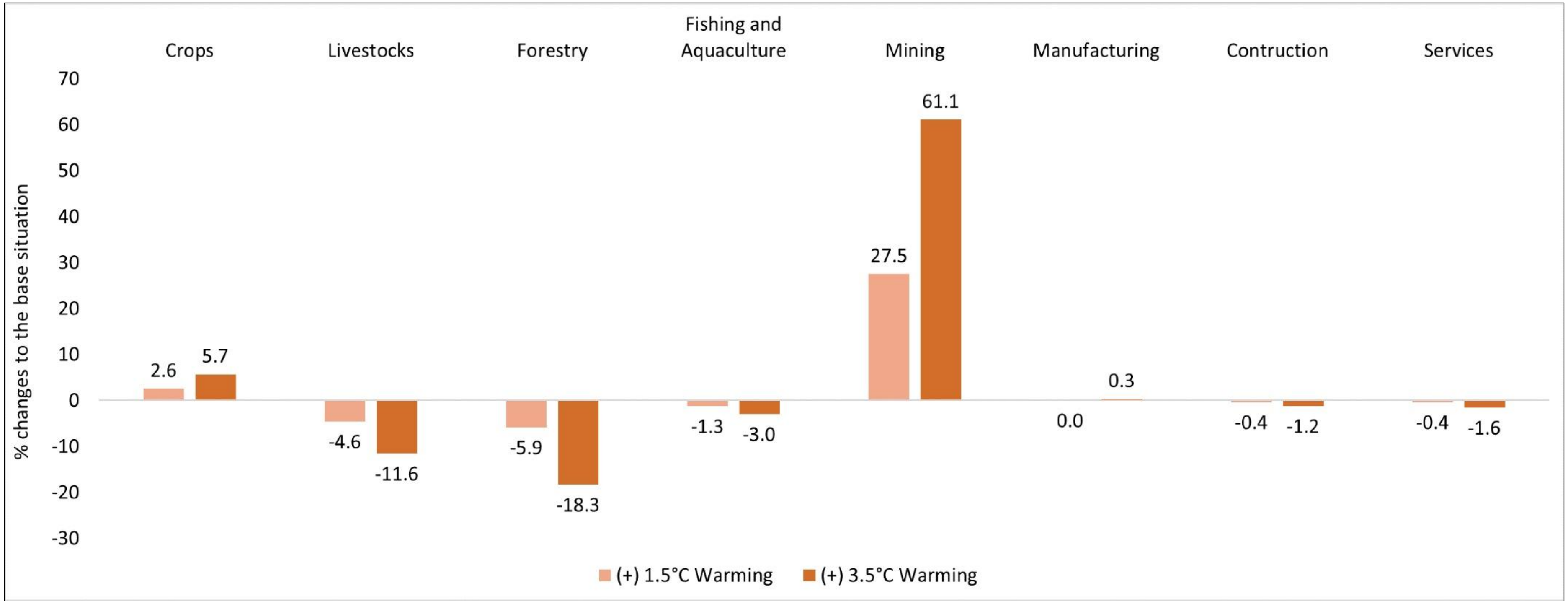

Supplementary Figure 6. The effects of heat stress-induced labour productivity loss on household expenditure

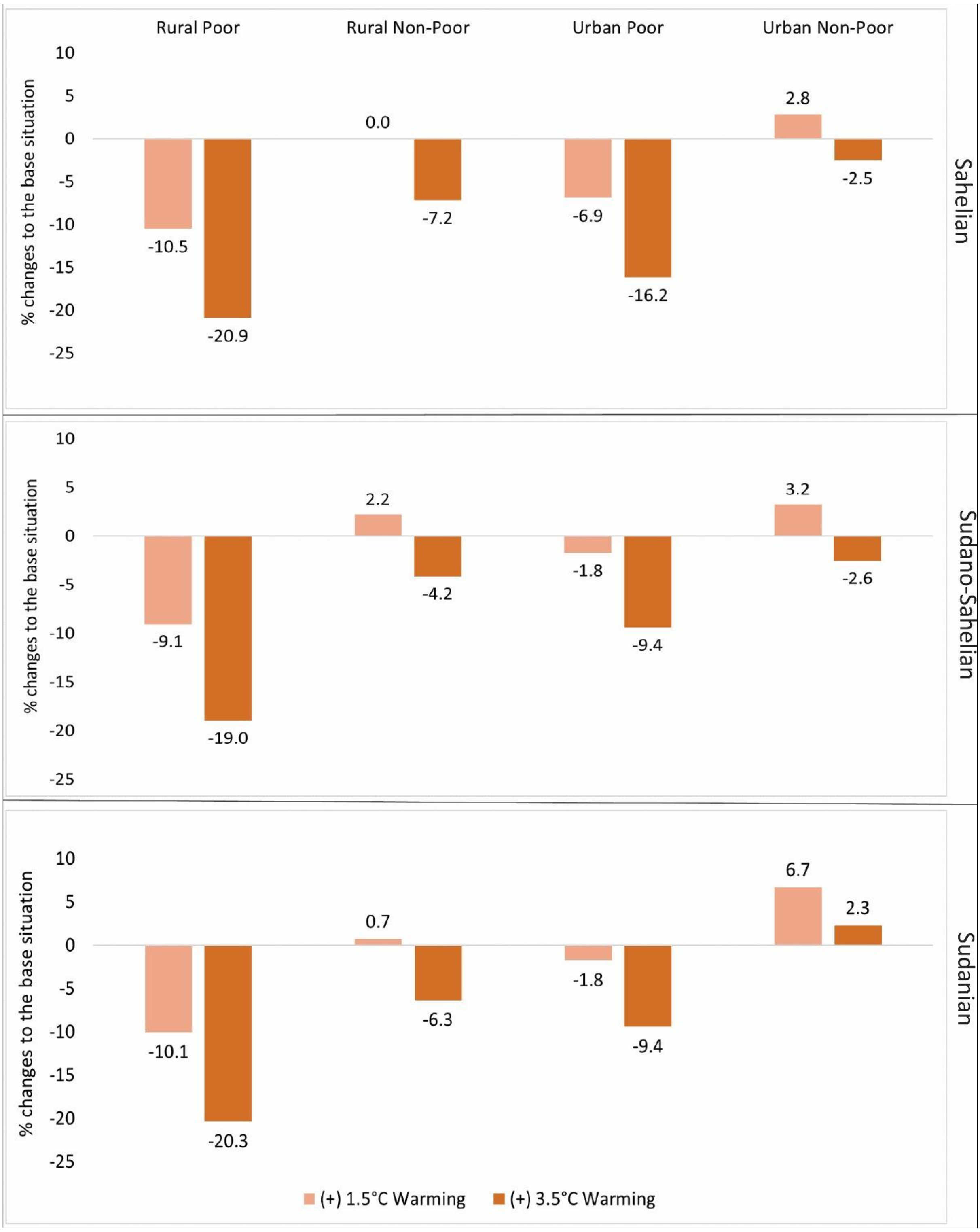

Supplementary Figure 7. The effects of heat stress-induced labour productivity loss on rural and urban household welfare changes across AEZs

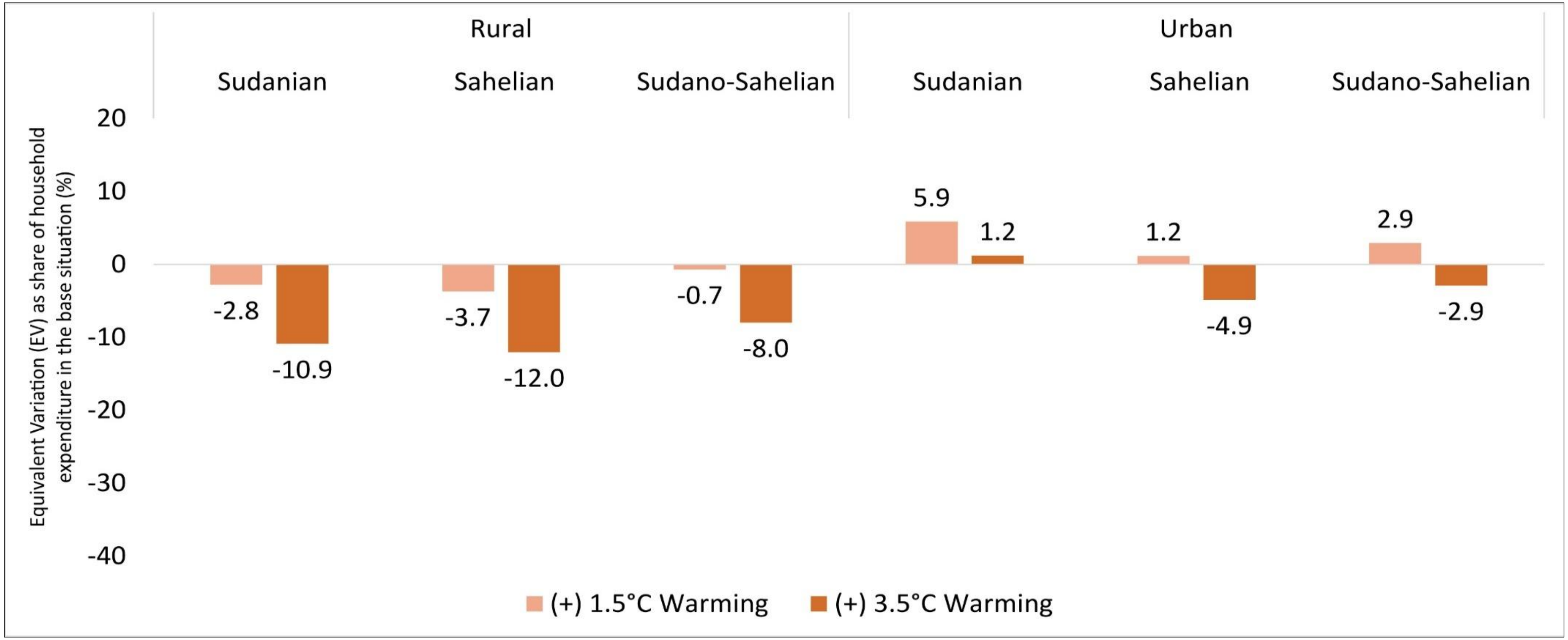

Supplementary Figure 8. Regions and agroecological zones in Burkina Faso

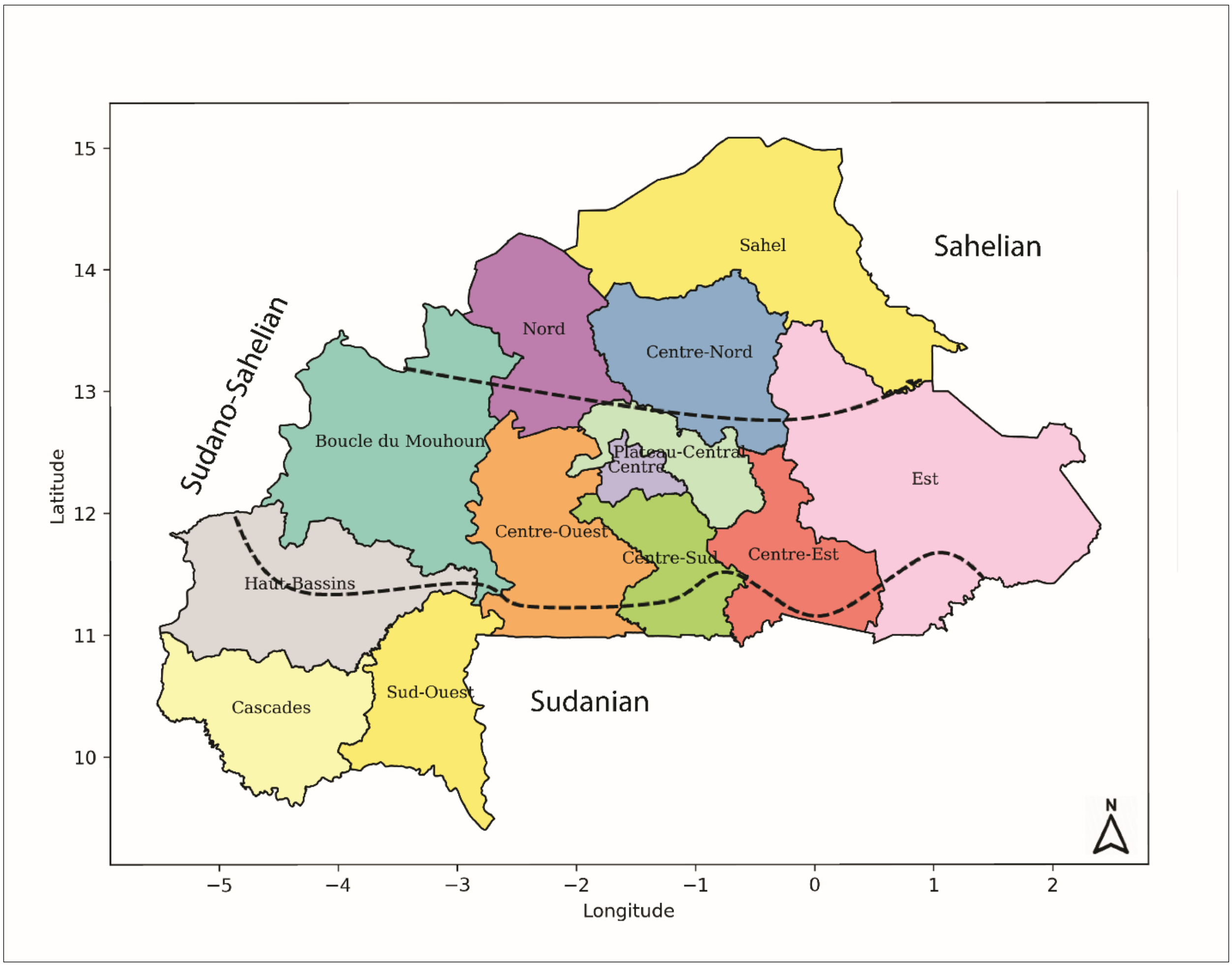

Note. The Figure shows the map of Burkina Faso, differentiating the region boundaries and agroecological zones delimited using dashed lines. We created the map in Python software using a publicly available shapefile for Burkina Faso.

Supplementary Figure 9. Production nesting in the STAGE model

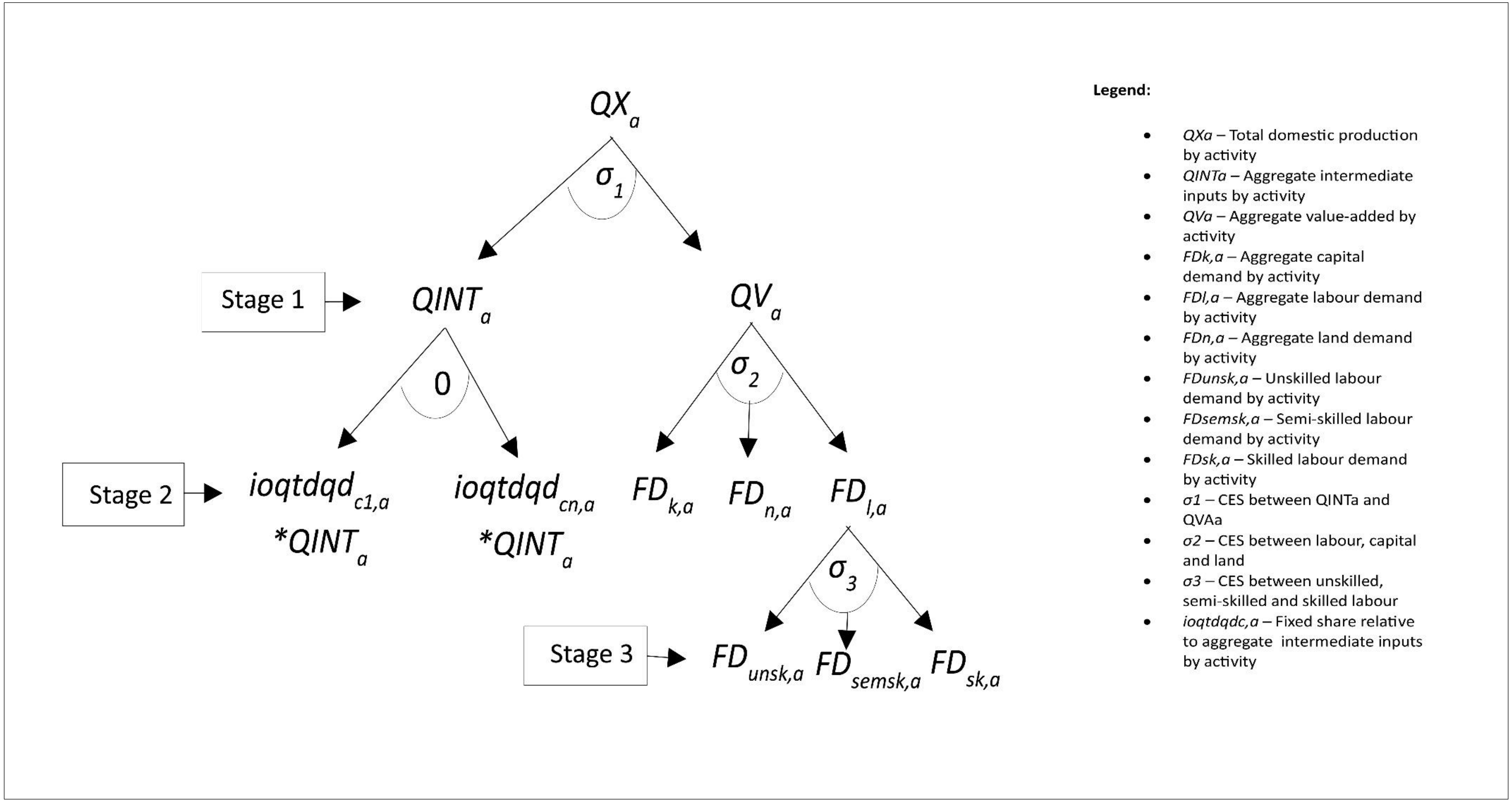

Note. The figure presents a standard production nesting in the STAGE model used for the study. We adapted from Ref.[60] (in Methods) and created it using Adobe AI software.

Supplementary Table 1. Household consumption composition (row share in %)

	Crop	Livestock	Fishing and Aquaculture	Forestry	Mining	Food Manufacturing	Non-Food Manufacturing	Construction	Services	Total
Rural Poor (Sudanian)	25.1	6.8	0.3	1.8	0.0	37.9	18.9	0.1	9.0%	100
Rural Poor (Sahelian)	24.8	6.8	0.3	1.8	0.0	38.1	19.0	0.1	9.0%	100
Rural Poor (Sudano-Sahelian)	21.0	7.0	0.2	1.5	0.0	40.0	20.0	0.1	10.2%	100
Urban Poor (Sudanian)	29.5	6.5	0.5	1.8	0.0	35.4	17.7	0.1	8.5%	100
Urban Poor (Sahelian)	29.6	6.6	0.5	1.8	0.0	35.3	17.6	0.1	8.5%	100
Urban Poor (Sudano-Sahelian)	25.6	6.9	0.3	1.9	0.0	37.3	18.6	0.1	9.2%	100
Rural Non-Poor (Sudanian)	14.9	6.8	0.3	1.7	0.0	31.2	26.3	0.1	18.7%	100
Rural Non-Poor (Sahelian)	14.9	6.8	0.3	1.7	0.0	31.2	26.3	0.1	18.7%	100
Rural Non-Poor (Sudano-Sahelian)	9.9	4.6	0.2	1.2	0.0	36.0	30.3	0.1	17.7%	100
Urban Non-Poor (Sudanian)	13.5	5.9	0.2	1.6	0.0	32.5	27.3	0.1	18.8%	100
Urban Non-Poor (Sahelian)	16.6	6.6	0.4	1.7	0.0	30.7	25.8	0.1	18.1%	100
Urban Non-Poor (Sudano-Sahelian)	8.5	4.7	0.2	0.7	0.0	32.3	27.2	0.1	26.3%	100
All households	13.0	5.5	0.2	1.2	0.0	34.1	26.3	0.1	19.7%	100

Source: Authors' compilation based on the developed 2019 SAM.

Supplementary Table 2. Heat stress-induced labour productivity loss based on the Lancet-LRF (in %)

	+1.5°C warming						+3.5°C warming					
	Unskilled		Semi-Skilled		Skilled		Unskilled		Semi-Skilled		Skilled	
	Lancet-LRF	ISO-LRF	Lancet-LRF	ISO-LRF	Lancet-LRF	ISO-LRF	Lancet-LRF	ISO-LRF	Lancet-LRF	ISO-LRF	Lancet-LRF	ISO-LRF
Agriculture (Sudanian)	-4.7	- 9.9	-4.7	- 9.9	-3.0	- 4.0	-14.3	- 26.6	-14.3	- 26.6	-10.0	- 16.7
Agriculture (Sudano-Sahelian)	-5.2	- 10.4	-5.2	- 10.4	-3.3	- 5.5	-15.0	- 26.9	-15.0	- 26.9	-10.8	- 19.6
Agriculture (Sahelian)	-5.5	- 10.8	-5.5	- 10.8	-3.5	- 6.1	-15.6	- 27.3	-15.6	- 27.3	-11.4	- 21.1
Mining	-5.2	- 10.4	-5.2	- 10.4	-3.3	- 5.4	-15.0	- 26.9	-15.0	- 26.9	-10.9	- 19.6
Manufacturing	-4.1	- 9.3	-4.1	- 9.3	-2.2	- 2.4	-12.5	- 25.6	-12.5	- 25.6	-7.9	- 13.5
Construction	-5.2	- 11.0	-5.2	- 11.0	-3.3	- 8.7	-15.0	- 26.9	-15.0	- 26.9	-10.9	- 24.3
Services	-2.2	- 7.1	-2.2	- 7.1	-0.9	- 0.3	-7.5	- 21.5	-7.5	- 21.5	-3.7	- 5.3

Supplementary Table 3. Changes in labour employment across sectors (in %)

	(+) 1.5°C Warming			(+) 3.5°C Warming		
	Unskilled	Semi-Skilled	Skilled	Unskilled	Semi-Skilled	Skilled
Crops	5.5	5.4	4.2	15.3	19.6	14.6
Livestock	13.5	13.3	11.9	32.3	37.1	30.9
Forestry	1.65	1.5	0.2	0.8	4.5	-0.2
Fishing and Aquaculture	17.9	17.7	16.3	48.0	53.5	46.8
Mining	-37.4	-37.4	-38.3	-56.8	-55.2	-57.3
Food Manufacturing	10.9	10.8	10.4	25.4	30.0	27.4
Non-Food Manufacturing	-2.0	-2.1	-6.0	-8.2	-4.8	-6.8
Construction	12.3	12.2	10.7	24.7	29.3	23.3
Services	0.2	0.1	-0.4	-4.8	-1.3	-1.4

Supplementary Table 4. Household factor income composition (row share in %)

	Unskilled Labour	Semi-Skilled Labour	Skilled Labour	Capital	Land	Total
Rural Poor (Sudanian)	84.1	4.7	1.8	3.4	5.9	100
Rural Poor (Sahelian)	89.8	4.2	0.3	0.8	4.9	100
Rural Poor (Sudano-Sahelian)	80.0	6.7	2.5	3.7	7.1	100
Urban Poor (Sudanian)	52.3	24.2	15.8	7.7		100
Urban Poor (Sahelian)	73.8	19.0	4.6	2.5		100
Urban Poor (Sudano-Sahelian)	48.3	27.5	17.4	6.8		100
Rural Non-Poor (Sudanian)	67.0	3.6	7.7	6.8	15.0	100
Rural Non-Poor (Sahelian)	79.9	2.4	1.8	1.9	14.0	100
Rural Non-Poor (Sudano-Sahelian)	69.8	3.3	11.2	5.2	10.5	100
Urban Non-Poor (Sudanian)	18.0	7.3	53.4	21.3		100
Urban Non-Poor (Sahelian)	51.0	11.2	27.1	10.7		100
Urban Non-Poor (Sudano-Sahelian)	12.1	8.0	67.2	12.8		100
All households	45.8	6.5	33.4	9.1	5.2	100

Source: Authors' compilation based on the developed 2019 SAM.

Supplementary Table 5. Macro-SAM for Burkina Faso for 2019 (in billion F.CFA)

	Commodities	Activities	Labour	Capital	Land	Households	Enterprises	Government	Sale tax	Production tax	Direct tax	Savings & Investment	Rest of the world	Total
Commodities		5672.3				5749.2		1925.7				2117.2	2617.7	18082.1
Activities	14306.9													14306.9
Labour		6379.2												6379.2
Capital		1821.1												1821.1
Land		390.0												390.0
Households			6371.0	674.6	390.0		163.4	197.0					139.3	7935.3
Enterprises				1146.5				112.6					701.5	1960.6
Government									848.9	44.3	907.4		232.5	2033.2
Sale tax	848.9													848.9
Production tax		44.3												44.3
Direct tax						334.6	572.7							907.4
Savings & Investment						1851.5	828.5	-202.2					-360.6	2117.2
Rest of the world	2926.3		8.2				395.9							3330.4
Total	18082.1	14306.9	6379.2	1821.1	390.0	7935.3	1960.6	2033.2	848.9	44.3	907.4	2117.2	3330.4	

Source: Authors' compilation based on the developed 2019 SAM.
